# Access to primary care for socio-economically disadvantaged older people in rural areas: A qualitative study

**DOI:** 10.1371/journal.pone.0193952

**Published:** 2018-03-06

**Authors:** John A. Ford, Rachel Turley, Tom Porter, Tom Shakespeare, Geoff Wong, Andy P. Jones, Nick Steel

**Affiliations:** 1 Department of Public Health and Primary Care, University of East Anglia, Norwich, United Kingdom; 2 Nuffield Department of Primary Care Health Sciences, University of Oxford, Oxford, United Kingdom; International University of Health and Welfare School of Medicine, JAPAN

## Abstract

**Objective:**

We aim to explore the barriers to accessing primary care for socio-economically disadvantaged older people in rural areas.

**Methods:**

Using a community recruitment strategy, fifteen people over 65 years, living in a rural area, and receiving financial support were recruited for semi-structured interviews. Four focus groups were held with rural health professionals. Interviews and focus groups were audio-recorded and transcribed. Thematic analysis was used to identify barriers to primary care access.

**Findings:**

Older people’s experience can be understood within the context of a patient perceived set of unwritten rules or *social contract*–an individual is careful not to bother the doctor in return for additional goodwill when they become unwell. However, most found it difficult to access primary care due to engaged telephone lines, availability of appointments, interactions with receptionists; breaching their perceived social contract. This left some feeling unwelcome, worthless or marginalised, especially those with high expectations of the social contract or limited resources, skills and/or desire to adapt to service changes.

Health professionals’ described how rising demands and expectations coupled with service constraints had necessitated service development, such as fewer home visits, more telephone consultations, triaging calls and modifying the appointment system.

**Conclusion:**

Multiple barriers to accessing primary care exist for this group. As primary care is re-organised to reduce costs, commissioners and practitioners must not lose sight of the perceived *social contract* and models of care that form the basis of how many older people interact with the service.

## Introduction

As people age, the need for access to health care increases. Preventative measures, such as screening, health checks and chronic disease monitoring, leave older people requiring even more health care [1–3]. Some policies to improve access, aimed at the whole population, have been criticised because they are not sufficiently targeted at those with highest health need [4]. Such policies may increase health care use by the worried well, increasing pressure on the system and counterproductively reducing access to those most in need. Previous research has found that older people, those in rural areas, and socio-economically disadvantaged groups are at particular risk of poor access and often have higher health need [5, 6]. By triangulating routine data sources, we estimate that there are about 316,000 socio-economically disadvantaged older people living in rural areas of England [7, 8].

As part of a programme of research [9], we undertook a realist review looking at how socio-economically disadvantaged older people in rural areas access primary care and found a lack of research for this specific group [10]. By synthesising studies with data relating to any key characteristic of this group (older, socio-economically disadvantaged people or rural residents), we proposed a range of personal, community and health care barriers. These occurred across the patient pathway—from being able to identify a health problem, to obtaining an appointment and getting to it. Other systematic reviews, looking at access to primary care in general, have identified similar barriers such as distance, waiting times, cost and social support [11, 12].

The evidence base looking at interventions to overcome these barriers is even more limited. Two major systematic reviews, looking at ways to improve access to primary care in the general population, advocate context-specific models [13] targeted at different levels of the health care system [12]. Whilst these are useful general recommendations, they do not provide detailed practical guidance for commissioners and policy makers or for specific population groups; partly due to a limited evidence base. A more focused understanding of the barriers faced by this group is needed to help develop and implement these targeted, context-specific interventions and policies. The perspective of health professionals is important because the quality of services ultimately depends on the local team [14]. In the United Kingdom National Health Service, this local team is made of primary care staff within a General Practice (GP) surgery and allied community health professionals who work with the GP surgery staff. Therefore, our aim is to qualitatively explore barriers to primary care for socio-economically disadvantaged older people in rural areas by both identifying the barriers and understanding how these barriers affect access.

## Methods

### Sample and recruitment

Interviews were undertaken with older people and focus groups with health professionals. Ethical approval was obtained from the University of East Anglia Faculty of Medicine and Health Sciences Research Ethics Committee (reference 20142015–43) before the research began and for each protocol amendment. Written informed consent was obtained prior to any data collection. Findings are reported in accordance with COREQ guidelines [15].

Participants who lived in a rural area, were over 65 years old and relied on financial support, such as means-tested pension credit, housing benefit or council tax support, were recruited for semi-structured interviews. We specifically did not use the term “benefits” in recruitment material because of socio-cultural connotations. Rurality was defined, using the Office for National Statistics definition, as settlements with less than 10,000 residents [16]. Those in residential care or unable to consent were excluded. We purposively decided to recruit participants through the community rather than primary care organisations to avoid any perception that the research was directly associated with their GP surgery. Our sampling framework aimed at avoiding oversampling of women (criterion: include at least three men), younger participants (criterion: include at least two participants over 80 years old) and people from the same practice (criterion: include participants from four different GP surgeries). Recruitment and data collection took place between August 2015 and January 2016.

The recruitment strategy evolved throughout the research because of low participation. In the first round of recruitment, three electoral wards in the English rural county of Norfolk, given its large population size and geographic area, were selected based on rurality, household deprivation and pension credit claimants. Posters and/or invitations were displayed in post offices, churches, shops, social clubs and parish newsletters, and this led to recruitment of one participant. For the second round of recruitment, we introduced a £20 shopping voucher, increased the number of electoral wards, amended the invitations and hand delivered approximately 300 invitations; leading to four additional participants. For the third round, the lead researcher (JAF) was interviewed on local radio and discussed the research at local Age UK and older people’s forums; leading to an additional two participants. In the fourth round, over four weeks across six pharmacies, about 300 invitations were placed in the pharmacy bags of patients over 65 years old and having their medications delivered; this generated a further eight participants at which data saturation was reached.

For four focus groups, rural health professionals were recruited through the NIHR Clinical Research Network (CRN). The CRN invited general practitioners (GPs) and practice mangers from research-active practices in Norfolk and the research lead of Norfolk Community Health Care to identify community health professionals. Health professionals, or their employing organisations, were reimbursed for their time and travel. None of the participants were known to the research team prior to participation, except for one GP who was employed part-time at the University of East Anglia. No participants who contacted the research team subsequently refused to participate or withdrew.

### Data collection

Participants chose to be interviewed at home (n = 12) or the University of East Anglia (n = 3) while the focus groups were held at the university. All focus groups and interviews followed individual topic guides (see supplementary online material). Interviews lasted about one hour, and focus groups two. Interviews and focus groups were audio-recorded and transcribed verbatim by an experienced transcriber at the University of East Anglia. Field notes were audio-recorded immediately after the interviews and focus groups and subsequently transcribed. No interviews were repeated.

The interviews and focus groups were conducted by a single male researcher trained in qualitative methods (JAF, qualifications MBChB MSc) whilst a second qualitative researcher (TP) observed two of the focus groups. JAF is a public health doctor, undertaking a PhD, but introduced himself as a researcher looking at access to primary care, not revealing his clinical background to participants unless specifically asked, which happened in one interview.

### Data analysis

We analysed the data using thematic analysis, based on the steps outlined by Braun and Clarke [17]. Briefly, this started with familiarisation of the data through repeatedly listening to the interviews and focus groups and reading the transcripts (JAF and RT). Codes were generated by identifying and labelling key sections of text which contained data on access to primary care by two researchers independently (JAF and RT). Similar codes were then independently grouped to form initial candidate themes by the same two researchers (JAF and RT). Themes were refined iteratively through repeated discussions of the data between the two coding researchers, the wider research team, colleagues within the department, and two patient representatives. QRS NVivo was used to code the data [18]. JAF has previously undertaken a realist review in this area [10] and had substantial knowledge of the topic; therefore a constant comparison approach [19] was used to avoid confirmation bias.

To ensure rigour and trustworthiness, we implemented the following measures: 1) coding and generation of initial themes was undertaken independently by two members of the research team (JAF and RT), 2) transcriptions were checked for accuracy by the researcher undertaking the interviews (JAF) and 3) themes were interrogated by the research team and patient representatives. We did not undertake member checking, so as to leave the data as it was captured during the interview.

## Findings

In total fifteen participants, six men and nine women, were interviewed with an average age of 78 and a range from 67 to 87 years old. Six of these participants were over the age of 80. All participants lived in Norfolk and were registered at ten different GP surgeries.

Each of the four focus groups had between three and five participants. There were 16 participants in total: three GPs, four practice managers, five senior community nurses, three district nurses and one community physiotherapist. Two focus groups included only GPs and practice managers, and two community nurses and the physiotherapist.

**Fig 1:**
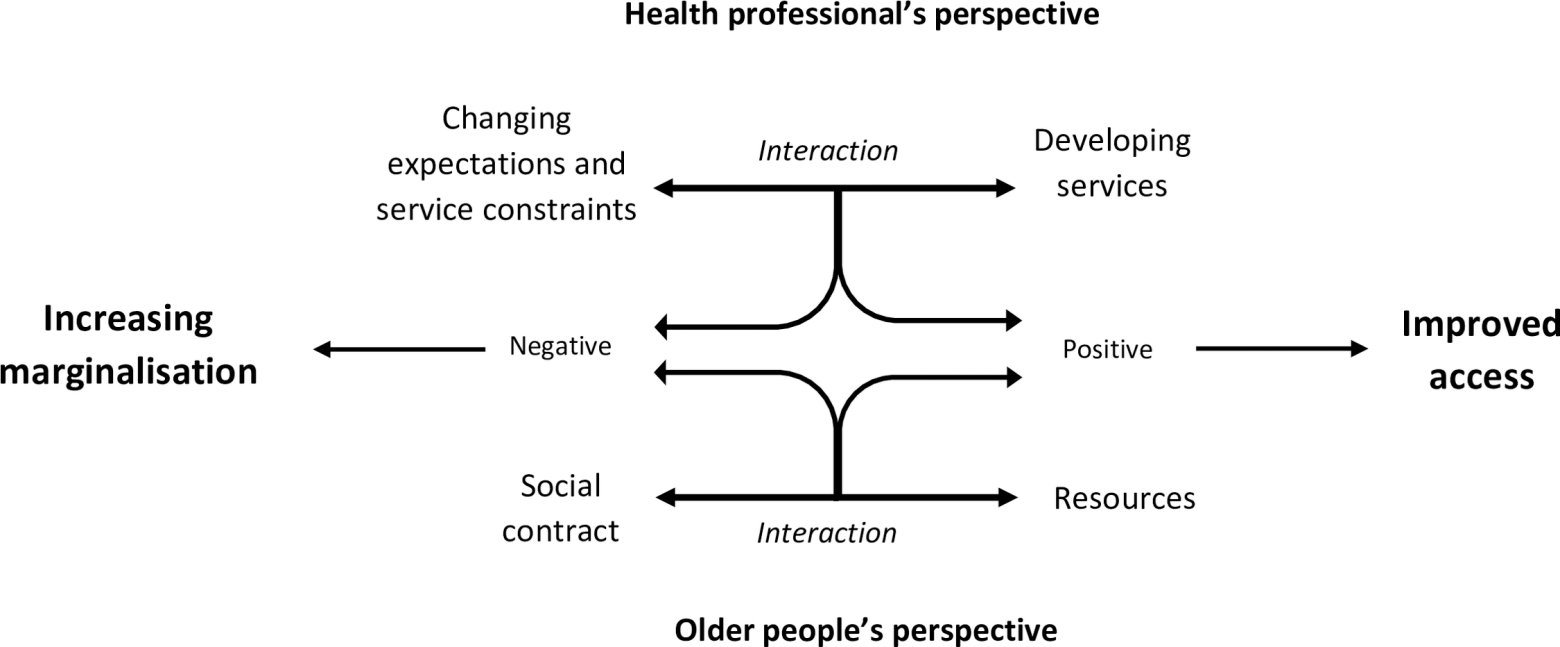
Older people and health professional’s perspective of accessing primary care.

The perspectives of older people and health professionals are presented, with participant quotes to illustrate each sub-theme. The demographics of interview participants are shown in Table 1, whilst Fig 1 shows a diagrammatic overview of the findings.

**Table 1:** Demographics of interview participants.

Participant number	Age	Gender	Housing type	Distance to GP surgery	Access to a car
1	75	Female	House	7.1 miles	Yes
2	82	Male	Sheltered accommodation	0.3 miles	No
3	83	Female	House	1.1 miles	No
4	73	Female	House	4.4 miles	Yes
5	74	Male	House	4.4 miles	Yes
6	67	Male	House	1.6 miles	No
7	77	Male	House	3.5 miles	Yes
8	76	Female	Sheltered accommodation	0.3 miles	No
9	75	Female	House	2.4 miles	Yes
10	85	Male	House	1.6 miles	No
11	85	Female	House	1.6 miles	No
12	87	Female	House	0.3 miles	No
13	75	Female	House	0.4 miles	No
14	82	Male	House	2.8 miles	No
15	79	Female	House	0.4 miles	

### Older people’s perspective

#### Social contract

Older people’s experience can be understood within the context of a patient perceived set of unwritten rules or *social contract*. These social norms are based on reciprocity–older people were careful not to “*bother*” or waste the doctor’s time, on the understanding that their GP surgery (doctors, nurses and receptionists) make special provision for them when unwell. Special provision meant goodwill, such as being flexible about rigid appointment systems or home visits. Participants saw this in the context of their whole life, insomuch as they expected the receptionists and GPs to be aware that during their lifetime they were not someone who inappropriately used the service. For example, this participant describes how, because she is careful not to ask for help unnecessarily, the GP does not question when she requests a home visit:

“I don’t come to a doctor unless I’m ill. When I say there’s something wrong with me [the GP] knows I don’t go there time wasting [because]… I only go if I’m really not feeling well… He’s never queried if we’ve wanted a call out, never been, “oh, can’t you come to the surgery”.” (Pt 13, 75 year old female)

Here the participant describes her perception that in return for fulfilling her responsibilities only to attend the GP surgery when necessary, the doctor is willing to undertake a home visit, even if he might query it with other patients. All participants discussed the importance of not bothering, or wasting the doctor’s time, unless absolutely necessary. Several participants considered how this attitude had changed in society throughout their life, such as this participant who compared a childhood event to current norms:

“When I was seven or eight the old man upstairs died because they wouldn’t send for a doctor on a Sunday… And this is the thing. I think a lot of people do go and bother, whether it’s doctors or hospitals or anybody else, about things which they shouldn’t be bothering them with.” (Pt 1, 75 year old female)

#### Breach of the social contract

When participants did decide to access primary care, most found it difficult despite being careful not to bother the doctor. Participants articulated a sense of frustration if the practice breached the social contract by not recognising that they were someone who used the service responsibly, thinking carefully before asking to see the doctor. For example, participants shared their dismay about asking for a GP appointment, only for a receptionist to ask what the symptoms were, or if it was something the nurse could have dealt with:

“I feel that’s rude. I feel like saying it’s nothing to do with you. My first reaction is, ‘it’s not your business. I need to speak to my GP. I’m ringing up about an appointment, I’m not ringing up to tell you what my symptoms are.’” (Pt 6, 67 year old male)

Here the participant expected the receptionist to recognise their carefully considered judgement of when to request a doctor’s appointment. In return they expected the receptionist not to question their decision.

Participants identified difficulties such as engaged telephone lines, availability of appointments, receptionists and home visits. One participant reported having to wait 40 minutes to get through to a receptionist, by which time there were no appointments. Most highlighted the need to negotiate an appointment and usually perceived receptionists as barriers. Several participants described being told “*very firmly*” what they could or could not do. One participant described the receptionists as *“gods” (Pt 7*, *77 year old male)*. Some participants described positive experiences of receptionists, but others described negative experiences:

“I get annoyed with them because I don’t feel that I’m ringing up and wasting their time. They are paid to do a job. They are paid to be polite.” (Pt 15, 79 year old female)

Another source of frustration, particularly for those without a car, was being unable to get a home visit or given a telephone consultation instead of a visit.

Some who prioritised this social contract or lacked the resources, skills and/or desire to adapt to service changes were left feeling marginalised; the perceived exclusion from services based on personal characteristics, in particular ageing. For example, this participant described how she felt when contacting the GP surgery, even after careful use of GP services over the course of her life:

“You just feel so unwelcome and a nuisance, such a nuisance.” (Pt 1, 75 year old female)

Other participants felt ostracised, unwelcome or worthless because they were not treated with the respect that they felt they deserved as older people and thoughtful users of the service, such as this participant:

“You feel that you’re not worth anything. You really feel that all right because you’re elderly you’re not wanted. Ok, curl up in the corner, die quietly. Just leave us alone.” (Pt 15, 79 year old female)

This highlights the additional emotional challenges that older people face accessing primary care.

#### Impact of physical resources and skills

Having a car was a key resource to being able to get to the GP surgery. Most participants with a car were concerned about what would happen when they could no longer drive, with one reporting that they were planning to move house. For those without a car, the availability of public or community transport was discussed, but most relied on taxis, friends or family. The support provided by an individual’s friends and family was important, not only for transport, but also to help with making appointments.

It became evident that personal skills, such as ability to communication effectively, were important to obtain an appointment. These were often associated with previous experiences, such as type of employment. One participant who had previously worked in a citizen’s advice role, described her personal strategy of negotiating with someone over the phone.

“As long as I never contradict anybody on the phone I’m fine because they’ve got feelings as well. I have to say I’m asking because I don’t know and not sort of apportion blame as to who did it [because] sometimes there isn’t a ‘who did it’ it’s just one of these things.” (Pt 8, 76 year old female)

Other participants described how conversations with receptionists could escalate and become confrontational.

One participant appeared to have the resources and skills to engage with the system, but not the desire, preferring to retain their existing way of life. For example, she acknowledged the need for a touch telephone to help book an appointment but did not want to conform to the current expectations:

“It just annoys me. These little things annoy me, so I sort of cling to my phone but I will have to obviously get one with buttons on at some point.” (Pt 1, 75 year old female)

### The health professional’s perspective

#### Health professionals’ views of older people’s expectations of the health service and ageing

The growing expectations of the public was discussed in all focus groups. Health professionals felt that the expectations and needs of the wider population were inconsistent with current patterns of service provision. For example one health professional described a difficult conversation with a patient who expected a routine GP visit after an operation:

“It was finding the words to explain that that’s how it is now, without saying that’s how it is now. She hadn’t had an operation for so many years and she’d only ever called her GP if she’d been unwell. And she automatically expected him to call… And that generation that’s the least she expected. So she was really upset.” (FG4, community nurse)

The health professional identifies two reasons for this patient’s expectations of a routine home visit after a major operation; first, the system had changed since previous illness and, second, the patient used the service sparingly. This example given by a health professional supports the concept of a perceived social contract from older people.

Conversely, some health professionals reported that many older people from lower socio-economic groups do not seek help because they attribute health problems to ageing, leading to delayed presentation and disengagement with primary care:

“A lot of them are just thinking they’re getting old, so when something doesn’t work as well as it used to it’s just getting old. They don’t think let’s go and get it fixed, let’s go and get it investigated. That’s life—your hearing gets a bit worse, your eyesight gets a bit worse. For them it’s expected, so they just don’t engage with us.” (FG1, GP)

One health professional felt that some from lower socio-economic groups often had lower expectations of the health service and their own health compared to the wider population:

“I’d say there’s another hard core who are very low service users. They don’t expect anything out of life and therefore they don’t expect anything out of the health service and they tend not to consult.” (FG1, GP)

The above quotes highlight how health professionals attribute some of the lack of engagement, if not most of it, to the patient rather than the health service.

#### Service constraints

Restricted resources within the health care system, both nationally and locally, was discussed, especially the perception that primary care was being asked to do more without resources to match. This had a direct impact on our group of interest because financial constraints necessitated service reorganisation away from traditional models of care, which are more likely to satisfy a social contract. Health professionals identified other factors which exerted pressure on the service and ultimately access for our population group, such as the increase in recommended preventative medications leading to the medicalisation of ageing and requirement for more health care:

“Someone wants them to have pills. And whether it’s statins or whatever. People are on pills because they are ill, that’s one thing, but we’ve created lots and lots and lots of patients who need pills. So they’re people that we have turned into patients. They’re not ill!” (FG2, GP)

National or regional policies, and enhanced services, added pressure to service delivery. Generally health professionals were cynical about these and did not feel that they improved services for disadvantaged older people. For example, policies attempting to promote continuity through a named GP were seen as “*box ticking exercises*” since appointments were rarely available.

Furthermore, several health professionals descried how different groups of older people used services, sometimes inappropriately. For example, one GP identified inequalities between healthy older people with good access and ill older people with poor access:

“I’ve seen the wealthy 70 something who’s making an appointment a week to come and show me some skin blemish that they’re worried about because they have too many holidays on Majorca or whatever. And then I’ve got other people who really struggle to access the service [because] they’ve got transport issues and so on and then when they come they’ve got a long list of things that need sorting out. It seems very inequitable to me.” (FG1, GP)

#### Need for targeted services for this group

Faced with increased demands and limited resources, health professionals identified the need to re-organise services to reduce costs. These changes often negatively impacted socio-economically disadvantaged older people. Changes included fewer home visits, more telephone consultations, triaging calls and modifying the appointment system, as described by this GP:

“Over the last four or five years we’ve reiterated our way of people accessing the system umpteen times. So we now have telephone appointments, we have telephone triage, on the day, open surgery system so there are no pre-booked, [and] a nurse practitioner service.” (FG1, GP)

However some re-organisation has led to opportunities. One GP suggested that an outcome of having so many ways of accessing primary care was an ability to accommodate the needs of different older people, such as those without a car. For example, the GP described how a universal approach may miss some groups and how his practice had attempted to address this by becoming more flexible:

“I think we tend to try and operate a one size fits all service that catches as many people as we can. But we always know that there will be people around the margins of that who fall outside what it’s designed to cope with….One of the things that we have learned over the last decade or so is that we just have to make our system as flexible as we can to take advantage of opportunities because if you have this very rigid system… then somebody says well I want to come to surgery but I can only come at 12 o’clock which is when all the GPs are out on visits.” (FG1, GP)

Several health professionals described the need for a targeted approach for vulnerable groups, such as disadvantaged older people. However, not all health professionals felt that this should necessarily be a proactive approach which may involve the GP surgery initiating contact with this group, but rather responsive when this group needed help.

Health professionals also identified the need to re-educate how older people use the service by explaining the benefits of attending the practice, rather than requesting a home visit, and discussing the frequency of appointments and legitimate reasons for seeing the doctor.

## Discussion

### Summary of principal findings

We found that rural, socio-economically disadvantaged older people’s experience of access to primary care can be understood within the context of a *social contract*–an individual is careful not to bother the doctor in return for additional goodwill when they became unwell. However when participants accessed primary care, most found it difficult due to engaged telephone lines, availability of appointments, interactions with receptionists; breaching their perceived social contract. This left some feeling unwelcome, worthless or marginalised, especially those with high expectations of this social contract or limited resources, skills and/or desire to adapt to service changes. From a health professionals’ perspective, barriers included rising demands and expectations, necessitating service development of traditional models of care with less resource. Service developments included fewer home visits, more telephone consultations, triaging calls and modifying the appointment system.

### Strengths and limitations

One strength of our design was collecting data from both service users and health professionals allowing different perspectives of access to primary care. However, we experienced challenges in recruiting this group of hard to reach older people. Recruitment was particularly challenging because we used a community recruitment strategy, compared to recruiting via GP surgeries. We did this so that participants would be less likely to perceive that the research was directly associated with their own GP surgery. However it made recruitment more difficult and to overcome this challenge we modified our recruitment strategy on three occasions. Our final method, which resulted in most recruits, was to use delivered pharmacy bags to recruit vulnerable older people. This recruitment method that may be applicable to a range of other studies. However, participants were recruited using this method may have been more likely to be engaged with primary care and housebound.

We included both men and women and anyone over 65 years old, but did not have sufficient data to explore the difference between older men and women [20, 21]. The age of participants ranged from 67 to 87 years old. Those belonging to the ‘baby boomer’ generation (52 to 71 years old) and ‘silent generation’ (72 to 93 years old) may have different life experiences and attitudes, but similarly we did not have the data to explore this.

Our main inclusion criteria to identify socio-economically disadvantaged older people was relying on financial support, acknowledging that financial deprivation is only one aspect of disadvantage. Despite receiving financial support, at the interview stage two participants did not appear to fit this group, and their data was interpreted with caution.

Furthermore we only undertook four focus groups of health professionals and while similar themes began to emerge in the third and fourth focus groups we were may not have reached full saturation.

Whilst our data came from one area of the UK, we do not have any reason to think that the concept of a social contract is generalizable to other parts of the UK, although the terms of the reciprocal relationship may be different. Other studies including older people identified similar themes of not wanting to bother the doctor [22] and there is no suggestion that our findings are unique only to our participants. Although areas with low population and GP workforce mobility, such as Norfolk, may facilitate a social contract more than inner city areas. Further research is needed to understand if a social contract exists in other health care systems, such as the US. Previous research from the US has found similar barriers, such as transport, lack of resources, limited health care supply and lack of understanding by doctors [23–27]. However cost of medical treatment was more important in the US compared to the UK because of different health care systems. The health professionals’ perspective is likely to be generalisable across high income countries because most health care systems are trying to address the challenges of an ageing population, rising demands, increasing costs and financial constraints.

### Comparison with other studies

Our findings support the previously described concepts relating to access to services by vulnerable groups of candidacy (*“the ways in which patient's eligibility for medical attention and intervention is jointly negotiated between individuals and health services*” [28]) and permeability (*“the ease with which people can use services” [28]*). Health professionals and older people both alluded to the permeability of services, especially concerning availability of appointments, telephone access and receptionists. We found a two-way, dynamic relationship, supported by previous literature [29], between older people’s service use affecting practice procedures, and practices informing older people how to best use the service.

The concept of a *social contract* has been applied to health care previously, but at a macro level [30, 31]. Cruess and Cruess argued that members of society expect the medical profession to provide the services of a healer in return for autonomy, trust, status, self-regulation and a functioning health care system [30]. Our findings support the existence of a *social contract*, but enacted at a relational level and for a specific population group.

Bentley undertook a qualitative study using mini-ethnography to explore access to health care for older people living in an English village [32]. The study found that culture, legitimacy and, as suggested in previous research [21, 33], a retained hierarchical medical model of health care are barriers to access for older people. The authors found that older people did not identify themselves as consumers. We did not find data to support a consumer model either, and our findings support a hierarchical model in which older people do not bother the doctor for minor ailments. Building on this, we found that in return for this hierarchical system older people expect the GP surgery to make special provision when they are ill.

Goodridge and colleagues looked at the experiences of patients with chronic respiratory illness in rural areas of Canada using semi-structured interviews [34]. The authors found several important facilitators to health care, such as distance, relationship with their family doctor, supportive local community and health knowledge. We found that the GP relationship and local social networks were important, but older people in our study highlight the importance of suitable transport options rather than the physical distance to the surgery.

### Meaning of findings and policy implications

In England, Clinical Commissioning Groups have a legal obligation to address health inequalities [35]. Access to services is a key policy area to address inequalities [36]. Policy decisions in relation to access to primary care are challenging because most health services operate within a fixed budget, and improving access may lead to increased patient expectations or unnecessary use. One-size-fits-all policies may increase access for the whole population, leading to increased pressure on the system and counterproductively worse access for marginalised groups—a targeted approach is hence needed [12, 13].

Our findings suggest that health professionals support a targeted approach to improving access when vulnerable older people use primary care services, but not necessarily a proactive approach which may, for example, involve outreach activities in the community. We identified various personal and structural resources needed to access primary care, with the most important being transport, social networks and personal skills. An individual’s access to structural and personal resources were linked with their ability to manage service changes. For example, some older people are unfamiliar with changing staff roles, such as the changing role of a receptionist to include triaging, as well as booking appointments.

A tension exists in the health professional’s views–on one side ageing is being medicalised, but conversely patients do not consult because they attribute their health problems to ageing. It is likely that the medicalisation of ageing, in part, relates to conditions with incentivised or nationally recommended policies, such as raised cholesterol or blood pressure. It may that the some patients are being contacted to meet incentivised prevention targets, which in turn leads to fewer appointments to fulfil the social contract.

To address the significant financial and workforce challenges within the NHS, there is a drive to deliver primary care within ever larger practices, such as networks, federations or super-partnerships [37]. The effect of these changes are unknown. Our findings suggest a tension between the expectations within a social contract and experiences of older people which were compounded by recent service changes. Reconfiguring services to deliver primary care at scale may lead to socio-economically disadvantaged older people in rural areas feeling more disconnected from primary care, because, if a social contract is present, the older person will continue to carefully use the service, but are unlikely to be shown the goodwill they expect in return.

The interface between patient and practice is crucial and many practices have been striving to improve this for a long time. The drive to deliver primary care at scale is likely to make this interface, in the absence of careful planning, less personal. Representative patient groups could help practices to improve the experience of older people. Simple measures may make a significant difference, such as helping practice staff understand the perspective of different patient groups to facilitate a more sensitive approach to the needs of vulnerable groups. This may mean, for some patients, receptionists not asking about a patient’s health problem, or having a lower threshold for home visits. A major obstacle is time and resources within primary care; without giving reception staff more time to answer calls and appointments to allocate, older people are likely to continue to have negative experiences.

More community support is needed in rural areas to help with transport, such as community car schemes. Closer collaboration between commissioners and local authorities may help to improve public transport to health care. Furthermore it may help to protect some appointments which coincide with public transport timetables or when there is limited taxi availability, such as during school drop off and pick up.

### Conclusion

We found that rural, socio-economically disadvantaged older people’s experience of access to primary care can be understood within the context of a *social contract*–an individual is careful not to bother the doctor in return for additional goodwill when they became unwell. However the perceived social contract was often breached when participants accessed primary care due to engaged telephone lines, availability of appointments, interactions with receptionists. This left some feeling unwelcome, worthless or marginalised, especially those with high expectations of this social contract or limited resources, skills and/or desire to adapt to service changes. From a health professional perspective, barriers included rising demands and expectations leading to the need to reconfigure services with less resource. As primary care is re-organised to reduce costs, commissioners and practitioners must not lose sight of the perceived *social contract* and models of care that form the basis of how many older people interact with the service.
